# bFGF-genetically modified bone marrow-derived mesenchymal stem cells promote tendon-to-bone interface healing by promoting chondrogenesis and regulating macrophage polarization

**DOI:** 10.3389/fbioe.2026.1921167

**Published:** 2026-09-16

**Authors:** Jun Chen, Ruimin Cheng, Zheng Jin, Tao He, Ruokun Huang, Ming Zhang, Zhenhua Zhu, Dan Yang, Zhenhua Fang

**Affiliations:** 1 Wuhan Fourth Hospital, Hubei Provincial Sports Medicine Center, Hubei Provincial Clinical Research Center for Orthopaedics, Hubei Key Laboratory of Sports Injury and Precision Therapy, Wuhan, China; 2 Department of Dermatology, Wuhan Hankou Hospital, Wuhan, Hubei, China; 3 Day Surgery Center, West China Hospital, Sichuan University, Chengdu, Sichuan, China

**Keywords:** basic fibroblast growth factor, bone marrow-derived mesenchymal stem cells, fibrocartilage regeneration, macrophages, tendon-to-bone healing

## Abstract

**Background:**

Rapid regeneration and functional recovery of the enthesis following tendon-to-bone interface (TBI) injury remains a significant challenge. This study aims to investigate the feasibility of leveraging bFGF genetically modified bone marrow-derived mesenchymal stem cells (BMSCs) to enhance tendon-to-bone interface regeneration.

**Methods:**

The proliferation and chondrogenic differentiation effects of BMSCs after bFGF treatment were firstly studies *in vitro*, which was followed by transfecting the BMSCs with lentiviral vector encoding bFGF (BMSC-bFGF). The immunomodulatory effects of bFGF transfected BMSCs on macrophage polarization were assessed as well. Next, BMSCs or bFGF-transduced BMSCs were surgically injected into the TBI to observe their effects *in vivo*. Macroscopic observation, histological assessment, and biomechanical analysis were correspondingly performed to evaluate the regenerative efficacy of tendon-to-bone healing in a rat model.

**Results:**

*In vitro* experiments demonstrated that the lentivirus transfection of bFGF into BMSCs enhanced the proliferation and chondrogenic differentiation of BMSCs. More importantly, the bFGF transfected BMSCs also possessed excellent immunomodulatory capacity, which modulated macrophage polarization by promoting the transition of M0 macrophages towards M2 phenotype, thereby creating favorable microenvironment for the fast recovery of TBI injury. Accelerated regeneration of TBI injury was evidently observed *in vivo*, and promoted natural fibrocartilage differentiation was intimately correlated with the therapy of bFGF-transfected BMSCs.

**Conclusion:**

This study demonstrated that the employment of bFGF-transfected BMSCs accelerated tendon-to-bone regeneration by regulation of immune responses and promotion of fibrocartilage formation, thereby improving its healing quality of TBI injury. These findings suggested a novel and promising strategy by transfecting the BMSCs with bFGF gene for the treatment of TBI injury.

## 1 Introduction

Tendon-to-bone interface (TBI) injury is a common injury in sports medicine, often leading to pain, dysfunction, and tremendous socio-economic burden (Sun et al., 2020). TBI is a complex, heterogeneous structure that functions to absorb stress during mechanical loading (Du et al., 2024). As we know, tendon-to-bone interface, namely, the enthesis, comprises a transitional zone of four distinct yet continuous tissue types: bone, the mineralized fibrocartilage, non-mineralized fibrocartilage, and tendon (Zhang et al., 2026). As fibrocartilage is a load-bearing tissue, the enthesis is prone to injury. However, due to its relative avascularity and the limited regenerative capacity of fibrocartilage, the injured TBI rarely achieves complete structural restoration (Derwin et al., 2018; Chen et al., 2026). Previous studies have shown that fibrous scar tissue replaces both the non-mineralized and mineralized fibrocartilage after the injury of TBI(Gao et al., 2022; Du et al., 2024; Zhang et al., 2026). Unfortunately, this disorganized scar tissue is unable to transmit or distribute mechanical forces from bone to tendon, thereby increasing the risk of re-rupture at the healed site (Du et al., 2024; Zhang et al., 2026). Therefore, achieving rapid and fully functional regeneration of the enthesis after TBI injury remains a critical challenge that urgently needs to be addressed.

However, recent advances in tissue engineering have opened up an intriguing new avenue for the treatment of TBI injury. To date, bone marrow mesenchymal stem cells (BMSCs) are known to possess multilineage differentiation potential, enabling their differentiation into osteoblasts, chondrocytes, and tenocytes under specific culture conditions (Gu et al., 2025). This inherent plasticity renders BMSCs particularly suitable for enthesis reconstruction, which necessitates the coordinated regeneration of both osseous and fibrocartilaginous tissues. Currently, BMSC-based fibrocartilage formation relies on *in vitro* chondrogenic pre-differentiation prior to implantation. This conventional differentiation strategy presents several critical limitations, including the requirement for additional culture steps, dependence on high-dose administration of costly growth factors such as transforming growth factor-β3 and dexamethasone, and the potential of cytotoxic effects on implanted cells before the exertion of therapeutic efficacy (Lee et al., 2010). Consequently, there is an urgent need to develop novel strategies that not only can shorten the culture duration and reduce growth factor consumption, but also enhance chondrogenic differentiation efficiency to ultimately facilitate functional fibrocartilage regeneration. In this context, gene transfection has emerged as a powerful approach enabling more sustained and physiologically relevant delivery of therapeutic proteins, and basic fibroblast growth factor (bFGF), a key regulator of cell proliferation and differentiation, has been proposed as a promising candidate for such applications (Tu et al., 2025).

As a member of the fibroblast growth factor (FGF) family, bFGF has been widely employed in tissue engineering strategies (Tu et al., 2025). However, its translational success in human trials has been limited compared to the promising observations in animal studies, where inherent instability and short biological half-life of bFGF are attributed to this discrepancy. In recent years, gene transfection of functional proteins has therefore gained attention as an alternative approach to deliver therapeutic proteins, because this approach can overcome the limitations mentioned above. As demonstrated, BMSCs can be transduced with the bFGF gene, where the overexpression of bFGF gene has been shown to promote osteogenic differentiation of BMSCs(Lennon et al., 2018; Chen et al., 2022). Nevertheless, few studies have investigated whether bFGF enhances the chondrogenic differentiation capacity of BMSCs. To address this gap, the present study aims to determine whether BMSCs transfected with the bFGF gene undergo chondrogenic differentiation *in vitro*, and whether the implantation of BMSCs overexpressing with bFGF gene can achieve robust fibrocartilage regeneration *in vitro*.

Compelling evidences indicate that the immune system, particularly macrophages, plays a dispensable role in tissue regeneration (Julier et al., 2017; Zhang et al., 2024). Following TBI injury, various cytokines and immune cells are present at the injury site, such as neutrophils, macrophages, dendritic cells, and lymphocytes. However, the coordinated regulation of the local inflammatory response is essential for improving the quality of TBI healing (Li D. et al., 2025). In recent years, immunomodulatory strategies, such as inhibiting excessive M1 macrophage activation or promoting M2 polarization, have shown promise in enhancing TBI repair outcomes (Song et al., 2024; Zhang et al., 2024). BMSCs possess notable immunomodulatory properties that can alleviate inflammation and promote tissue regeneration, rendering them valuable for repairing injured tissues (Zou et al., 2024). However, the interaction between BMSCs and the immune microenvironment during TBI healing has received relatively little attention. To address this gap, the present study aimed to investigate the effect of BMSCs on TBI healing in a rat model.

## 2 Materials and methods

### 2.1 Isolation and characterization of BMSCs

Primary BMSCs were isolated from 3-week-old SD rats and cultured with a previously described method (Chen et al., 2022). In brief, rat femur and tibia were rinsed with phosphate-buffered saline (PBS), bone marrow was then flushed from bone cavities with culture medium. Obtained cells were seeded in a T25 culture flask and cultivated at 37 °C in an atmosphere containing 5% CO_2_ after centrifugation and resuspension. Culture medium was refreshed every 2 days, and cells were passed when BMSCs reached 80%–90% confluence. Rat BMSCs of the third passage (P3) was used for subsequent experiments. The multilineage differentiation potential and expression of cell surface antigens were analyzed to confirm the characterization of BMSCs as described by previous protocols (Chen et al., 2022).

### 2.2 CCK-8

P3 BMSCs were detached and seeded into 96-well plates at a cell density of 2000 cells/well, with three replicates in each well. After attachment, the cells were treated with various concentrations of bFGF (0, 5, 10 and 20 ng/mL), and consecutively cultivated for 9 days. Cell proliferation was measured by using a Cell Counting Kit–8 (CCK-8; Dojindo, Japan) according to the manufacturer’s instructions, and the absorbance at 450 nm was recorded using a spectrophotometer (Bio-Tek Instruments).

### 2.3 Chondrogenic differentiation of bFGF *in vitro*

P3 BMSCs were seeded into six-well plates at a cell density of 1.5 × 10^5^ cells in each well with three replicates. BMSCs were cultured with chondrogenic differentiation complete medium with supplementation of different concentrations of bFGF (0, 5, 10 and 20 ng/mL). The medium was changed every 2 days. After 21 days of consecutive culture, the cultured cells in each group were taken for subsequent detection.

#### 2.3.1 Cellular immunofuorescence (IF)

The cultured cells were fixed with 4% paraformaldehyde for 15 min and washed with PBS for 3 times. 0.5% Triton X-100 was added to permeabilize the cellular membrane at room temperature for 15 min. After washing with PBS for 3 times, cells were blocked with 10% goat serum for 1 h at room temperature. Primary antibody of rabbit-anti-rat Collagen type II (ab307674, Abcam) was incubated overnight at 4 °C. The next day, cells were incubated with Alexa Fluor 488-conjugated secondary antibody for 1 h at room temperature, and 4′, 6-diamidino-2-phenylindole (DAPI) was used to counterstain the cell nuclei after washing with PBS for 3 times. The immunofluoresence of Collagen type II were observed and photographed under a fluorescence microscope (Nikon, Japan), followed by analyzing with ImageJ software 1.8.0.

#### 2.3.2 Western blotting

Total cellular proteins were extracted using RIPA lysis buffer (Boster, China, AR0105-30), and the protein concentrations were determined with a BCA Protein Assay Kit (Pierce). The proteins were then separated by SDS-PAGE and transferred onto polyvinylidene fluoride (PVDF) membranes. The membranes were blocked with 5% non-fat milk for 1 h, followed by incubation with primary antibodies overnight at 4 °C. The primary antibodies used were against Sox 9 (Abcam, ab185966), Collagen II (Abcam, ab34712), and β-actin (Abcam, ab8227), with β-actin serving as the loading control. On the following day, the membranes were incubated with fluorescently labeled secondary antibodies for 2 h at room temperature. Protein bands were visualized using Quantity One software (Bio-Rad, Hercules, United States). The results were normalized to β-actin, and semi-quantitative analysis was performed using ImageJ software.

### 2.4 Chondrogenic evaluation after lentiviral transfection of bFGF

BMSCs were transfected with a bFGF lentiviral vector (Shanghai Jikai Gene Chemical Technology Co., Ltd.) according to the manufacturer’s instructions. To investigate the chondrogenic effect of bFGF on BMSCs, lentiviral vectors encoding either the bFGF gene (LV-bFGF) or a nonsense control sequence (LV-NC) were introduced into the cells across a range of multiplicities of infection (MOIs). The optimal MOI was determined and BMSCs were transduced at the optimal MOI, where transduction efficiency was measured by green fluorescent protein (GFP) expression. Cell proliferation was evaluated using a CCK-8 assay to generate a growth curve. The expression levels of bFGF mRNA and protein were detected by quantitative real-time reverse transcription PCR (qRT-PCR) and Western blotting, respectively. Total RNA extraction and qRT-PCR were performed as previously described, with the primers listed in Table 1 and GAPDH used as the internal control. Protein extraction and Western blotting were conducted as described in Section 2.3.2. Primary antibodies used were against bFGF (Abcam, ab92337) and β-actin (Abcam, ab8227).

**TABLE 1:** PCR primer for genes associated with chondrogenic differentiation.

Gene	Primer sequence	
bFGF	Forward primer	5′- GTG​TTA​CGG​ATG​AGT​GTT​TCT -3′
	Reverse primer	5′- CAG​CTC​TTA​GCA​GAC​ATT​GG -3′
GAPDH	Forward primer	5′- TGG​CTA​CAG​CAA​CAG​GGT​G -3′
	Reverse primer	5′- ATG​GCA​ACT​GTG​AGG​AGG​G -3′

To further investigate the induction of chondrogenesis following gene transfection, BMSCs transduced with lentiviral-bFGF were cultured in chondrogenic induction medium, with the medium refreshed every 3 days. Immunofluorescence staining for Collagen II was performed as described above. Observations were photographed under a fluorescence microscope (Nikon, Japan), followed by analyzing with ImageJ software of version 1.8.0. Concurrently, chondrogenic differentiation was assessed using Alcian blue staining.

### 2.5 Evaluation of immunomodulatory properties of BMSCs

The RAW 264.7 cells were procured from the China Center for Type Culture Collection and subsequently cultured under standard laboratory conditions in our facility. To investigate the effect of BMSCs on macrophages, the morphology and polarization changes of RAW 264.7 cells were examined under different stimulation conditions. The M1 positive control was induced with 100 ng/mL lipopolysaccharide (LPS, Sigma-Aldrich, United States), while the M2 positive control was induced with 20 ng/mL interleukin-4 (IL-4, Proteintech, United States). A transwell chamber (pore size 0.4 μm; BD Falcon, United States) was placed into a 24-well plate to establish an indirect co-culture system. 5 × 10^4^ BMSCs or bFGF-transfected BMSCs in 250 μL culture medium were seeded into the upper chamber, while 2.0 × 10^5^ RAW264.7 cells in 600 μL were cultured in the lower well plate. After 48 h of induction, the morphology of RAW264.7 cells was observed to assess cellular morphology changes under light microscope. To determine macrophage phenotype polarization, the expression of iNOS and CD206 was subsequently examined as prototypical markers for M1 and M2 subtypes, respectively. In addition, Western blotting and flow cytometry were also performed to analyze the protein expression of M1 and M2 markers, and primary antibodies of anti-iNOS (Abcam, ab178945), anti-CD206 (Abcam, ab64693), and anti-CD86 (Abcam, ab238468) were used in this study.

### 2.6 Animal experiments

The experimental protocol involving Sprague-Dawley (SD) rats was approved by the Animal Ethics Committee of West China Hospital, Sichuan University (20211192A). Male SD rats weighing approximately 300 g were used to establish tendon injury model. Prior to the procedure, SD rats underwent anesthesia and aseptic preparation of the surgical site. For preemptive analgesia, buprenorphine was administered subcutaneously at a dosage of 0.1 mg/kg body weight 1 hour before anesthesia induction. Induction was performed in a closed chamber with 5% isoflurane delivered in oxygen at a flow rate of 1 L/min. The endpoint of induction was defined as the complete loss of the righting reflex. Subsequently, the SD rats were relocated to the surgical platform, where anesthesia was maintained via a nose cone apparatus with the isoflurane concentration reduced to 1%. Throughout the procedure, respiratory rate was continuously monitored, and normothermia was preserved using a heating blanket. The Achilles tendon was sharply transected at its insertion footprint above the calcaneus, and all attached fibrocartilage at the insertion was removed. Bone tunnels were then created in the posterior calcaneus using a 0.8 mm drill, and a 6–0 silk suture was passed through the tunnel to secure the distal Achilles tendon to the calcaneus. The rats were randomly divided into three groups (n = 5 for each group). According to the treatment received. Postoperatively, the animals were permitted free cage activity and euthanized at 4 and 12 weeks for tissue harvest.

#### 2.6.1 Histological evaluation

The rats were administered a subcutaneous injection of buprenorphine (0.1 mg/kg body weight) at the 4- and 12-week endpoints. Following a 10-min equilibration period, the animals were humanely euthanized via cervical dislocation performed by experienced technicians. The tendon-to-bone junction (TBJ) tissues were harvested, fixed in 4% paraformaldehyde, decalcified in 10% ethylenediaminetetraacetic acid (EDTA) for 4 weeks. Harvested samples were then dehydrated in graded ethanol series, embedded in paraffin wax, and sectioned into 5-μm thick serial sections along the longitudinal plane. For routine histological evaluation, sections were stained with hematoxylin and eosin (H&E). To assess fibrocartilage formation at the tendon–bone interface, sections were stained with toluidine blue (TB) and Safranin O-Fast Green (SOFG). The area of fibrocartilage was measured using ImageJ software, with positive staining area normalized to the total interface region. Picrosirius red staining was used to evaluate collagen organization and maturation, where reddish-yellow coloration indicated dense, mature, and well-aligned collagen fibers, while green coloration represented thin, loosely arranged, and immature collagen fibers. All histological analyses were performed in a blinded manner by two independent observers.

#### 2.6.2 Biomechanical testing

To further evaluate the healing quality of TBI after the surgical reconstruction, we performed biomechanical tests at 4 and 12 weeks after the operation using a mechanical testing system ((Instron 6800 System, United States), and the testing protocols were based on the manufacturer’s instructions. In brief, tendon-to-bone samples were fixed firmly on the mechanical testing system, specimens were stretched at a speed of 5 mm/min until gross failure of the reconstructed TBI occurred, where the maximum load was recorded. The stiffness was calculated based on the linear portion of the load-deformation curve.

### 2.7 Statistical analysis

GraphPad Prism 10.0 software packages was used for all statistical analyses. All parameters were presented as mean ± standard deviation, and Student’s t-test or One-way analysis of variance (ANOVA) with *post hoc* Tukey’s test was used to determine the inter-group differences, and *p < 0.05* was considered significant.

## 3 Results

### 3.1 Isolation and identification of BMSCs

BMSCs were successfully isolated from the rat bone marrow cavity. Under light microscopy, the BMSCs displayed a spindle-like morphology (Figure 1A). To evaluate their multilineage differentiation potential, the cells were cultured in specific induction media, and tri-lineage differentiation ability was confirmed using Oil Red O, Alizarin Red, and Alcian blue staining, respectively (Figure 1A). Flow cytometric analysis was also performed to assess the expression of surface markers. The results demonstrated high expression of the stem cell markers CD90 and CD105, whereas the cells were negative for CD45 (Figure 1B).

**FIGURE 1:**
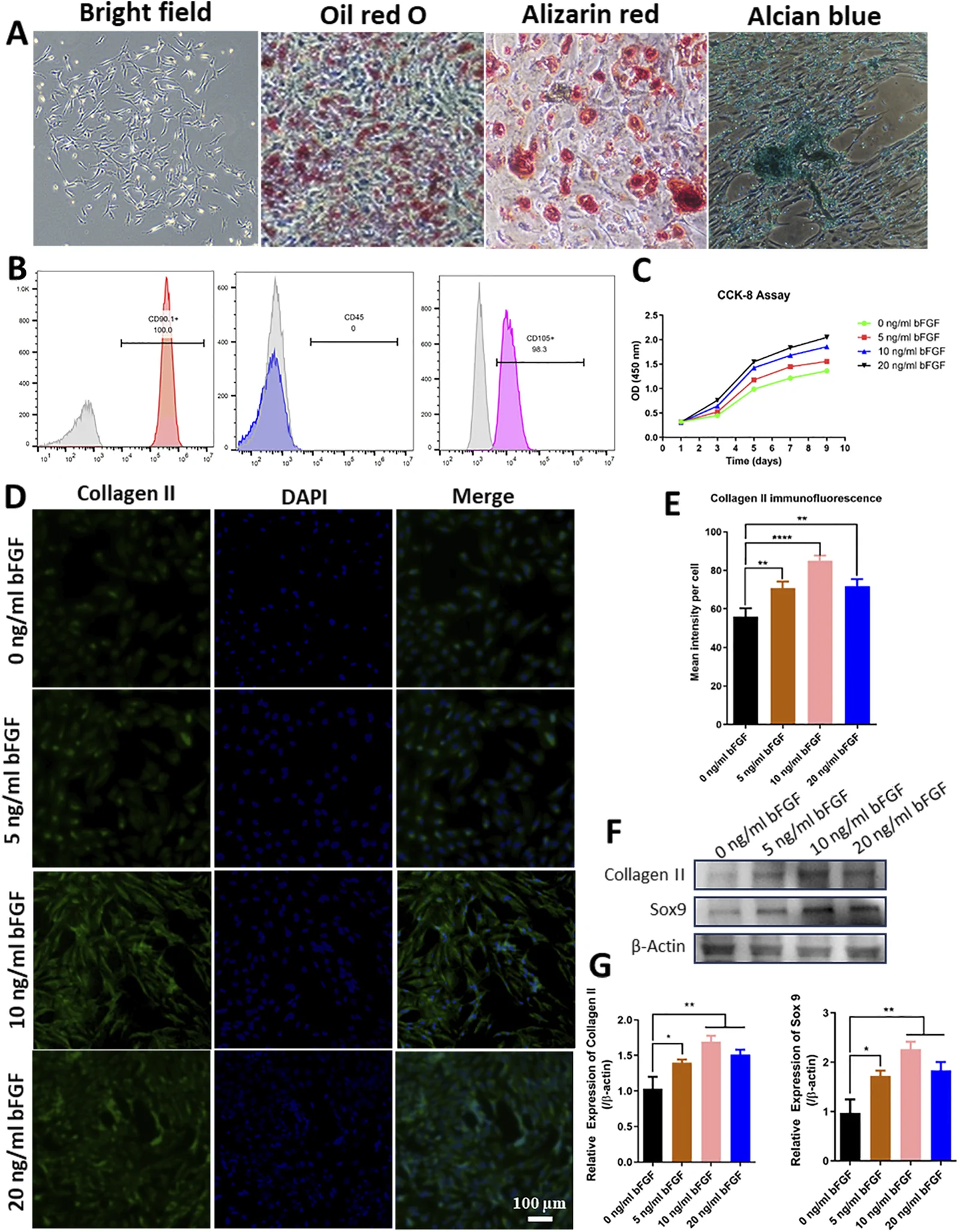
Morphology and characterization of BMSCs and the influence of bFGF on the BMSCs proliferation and chondrogenic differentiation. The identification of osteogenic, adipogenic, and chondrogenic differentiation potential of BMSCs. **(A)** Scale bars = 100 μm. Flow cytometry analysis of the expression of BMSCs surface markers. **(B)** The proliferative curve of each group. **(C)** Immunofluorescence **(D)** and mean fluorescence intensity statistics **(E)** of Collagen II, Scale bar = 100 μm. The protein levels of Sox 9 and Collagen II were detected by Western blotting **(F)** and quantification with inter-group comparison. **(G)** Data are presented as mean ± SD. Significant differences are indicated as *p < 0.05, **p < 0.01, ***p < 0.005, ****p < 0.0001. NS, not significant.

### 3.2 Proliferation and chondrogenic induction performance of BMSCs by bFGF

Following exposure to varying concentrations of bFGF, the proliferative capacity of BMSCs was assessed using the CCK-8 assay. The resulting growth curves exhibited a sigmoidal pattern across all groups (Figure 1C). The proliferation rate of BMSCs in the 5 ng/mL bFGF group showed no significant difference compared to the control group. By contrast, the proliferative capacity of the 10 ng/mL and 20 ng/mL bFGF groups was significantly higher than that of the control group (*p* < 0.05).

After 21 days of induction with bFGF, immunofluorescence staining for collagen type II was performed to evaluate chondrogenic differentiation of BMSCs. As shown in Figures 1D,E, collagen II expression was significantly upregulated in all treatment groups compared to the control group. Notably, cells cultured with 10 ng/mL bFGF exhibited the highest expression of collagen type II compared with those groups treated with 5 ng/mL or 20 ng/mL bFGF. The protein expression levels were further examined by Western blotting, which results were consistent with the immunofluorescence findings (Figures 1F,G). Collectively, these results indicated that the addition of bFGF evidently enhanced the chondrogenic differentiation of BMSCs, and 10 ng/mL bFGF was validated to be the most effective concentration for promoting this differentiation.

### 3.3 Stable expression of the bFGF gene and protein after bFGF gene transfection

BMSCs were transfected with lentivirus at various multiplicities of infection (MOIs). The percentage of GFP-positive BMSCs was assessed under a fluorescence microscope 72 h post-transduction (Figure 2A). An MOI of 75 yielded the highest GFP-positive rate and was therefore selected for subsequent experiments. Cell viability following lentiviral gene transduction was evaluated using the CCK-8 assay. As shown in Figure 2B, transfection with Lentiviral-GFP or Lentiviral-bFGF had no influence on cell viability. bFGF expression of BMSCs after transfection were detected by qRT-PCR and Western blotting. Accordingly, the qRT-PCR results revealed that bFGF mRNA expression in the lentivirus-transfected group was significantly higher than in the plasmid vector group and the untransfected control group (*p* < 0.05, Figure 2C). No significant difference was observed between the plasmid vector group and the untransfected control group (*p* > 0.05, Figure 2C). Of note, similar expression trends were observed by Western blotting, consistent with those obtained from qRT-PCR (Figures 2D,E), demonstrating successful transfection of bFGF into the BMSCs.

**FIGURE 2:**
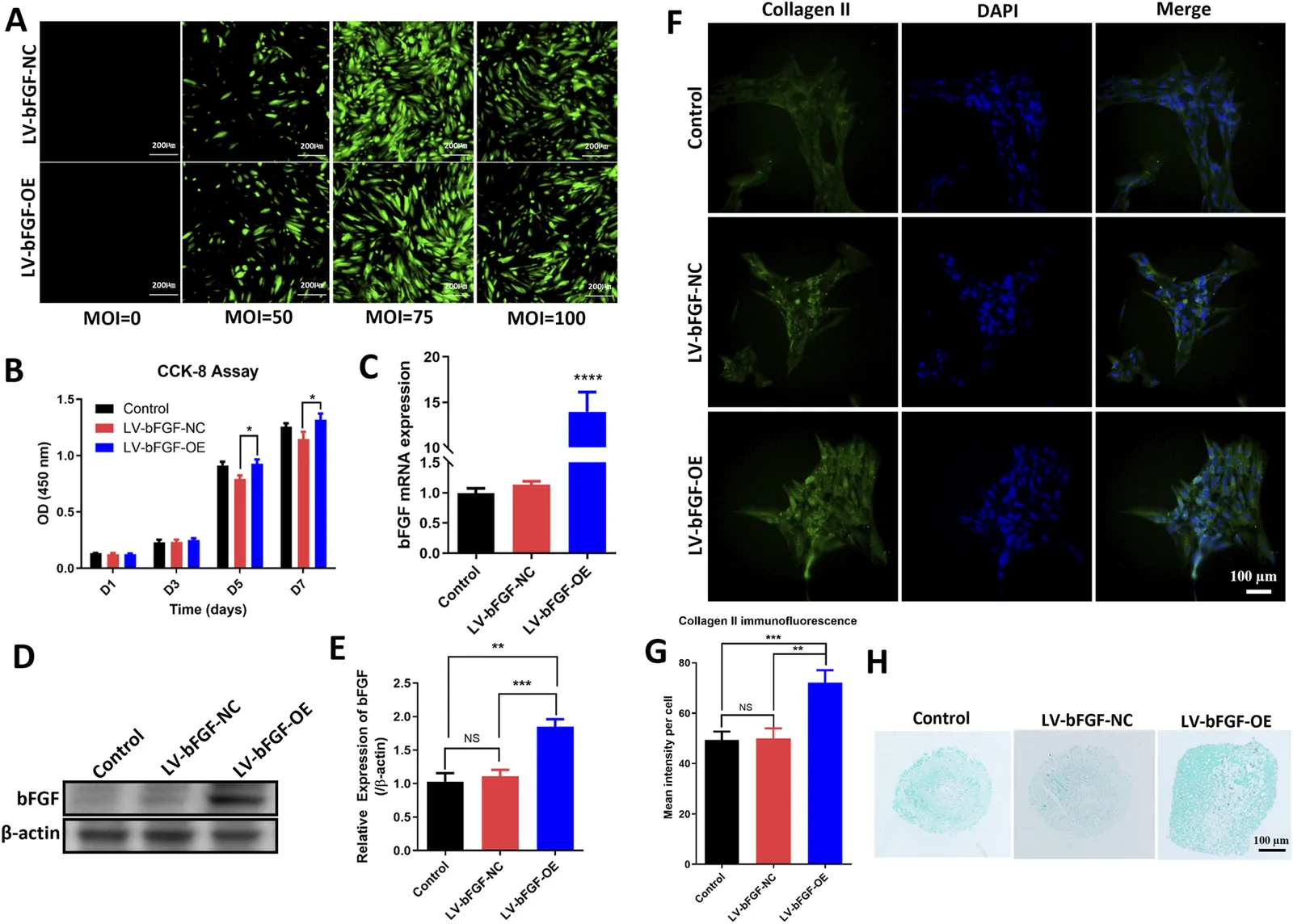
Lentiviral-bFGF transduced BMSCs and *in vitro* chondrogenic differentiation of lentivirus-transduced BMSCs. BMSCs with Lentiviral-bFGF vectors at different MOIs. **(A)** Scale bars = 200 μm. Cell proliferation assays were performed at 1, 3, 5 and 7 days post-transduction. **(B)** mRNA and protein expression of bFGF after transfection. **(C–E)** Immunofluorescence **(F)** and mean fluorescence intensity statistics of Collagen II **(G)** Scale bar = 100 μm. Alcian blue staining of cartilage pellets cultured with chondrogenic induction medium. **(H)** Data are presented as mean ± SD. Significant differences are indicated as *p < 0.05, **p < 0.01, ***p < 0.005, ****p < 0.0001. NS, not significant.

### 3.4 Chondrogenic induction performance after bFGF transfection

To further evaluate the chondrogenic differentiation capacity of BMSCs after the transfection of bFGF gene, immunofluorescence was conducted to detect the expression of collagen type II, a typical chondrogenic marker. As shown in Figures 2F,G, the expression of collagen type II was significantly higher in bFGF-transfected BMSCs than that of the control group or Lentiviral-GFP transduced BMSCs. 28 days post-transduction, Alcian blue staining revealed that cell pellets from the Lentiviral-bFGF group exhibited stronger staining compared with those of the control group and Lentiviral-GFP group (Figure 2H). Taken together, these results demonstrated that the transfection of BMSCs enhances the chondrogenic differentiation capacity of BMSCs.

### 3.5 Immunomodulatory property of BMSCs transfected with bFGF

The indirect co-culture system was shown in Figure 3A. The morphology of round shape was observed in the unpolarized RAW 264.7 macrophages, while the morphology could be changed when inducing agents were added. As shown in Figure 3B, macrophages exhibited different morphologies under different stimuli in the LPS and IL-4, where round shape with thorny tentacles in the LPS group and elliptical or fusoid shape in the IL-4 group were detected (Figure 3B). Interestingly, after co-cultured with BMSCs or bFGF transfected BMSCs, cellular morphology of macrophages were more similar to that of IL-4 groups. Further, Western blotting analysis revealed that the highest iNOS expression was detected in the LPS group, while CD206 expression was higher in the IL-4, BMSCs, and bFGF transfected BMSCs groups, compared with the control and LPS groups (*p* < 0.05), whereas the highest expression was observed in the IL-4 group (Figures 3C–E). Moreover, flow cytometry showed that higher percentage of CD206-positive cells was detected in the BMSCs and BMSC-bFGF groups compared to the control and LPS groups, even the positive ratios in those two groups were slightly lower than that of the IL-4 group (Figures 3F,G). Collectively, these results validated that both BMSCs and BMSC-bFGF had the capacity to promote the polarization of M0 macrophages towards the M2 phenotype.

**FIGURE 3:**
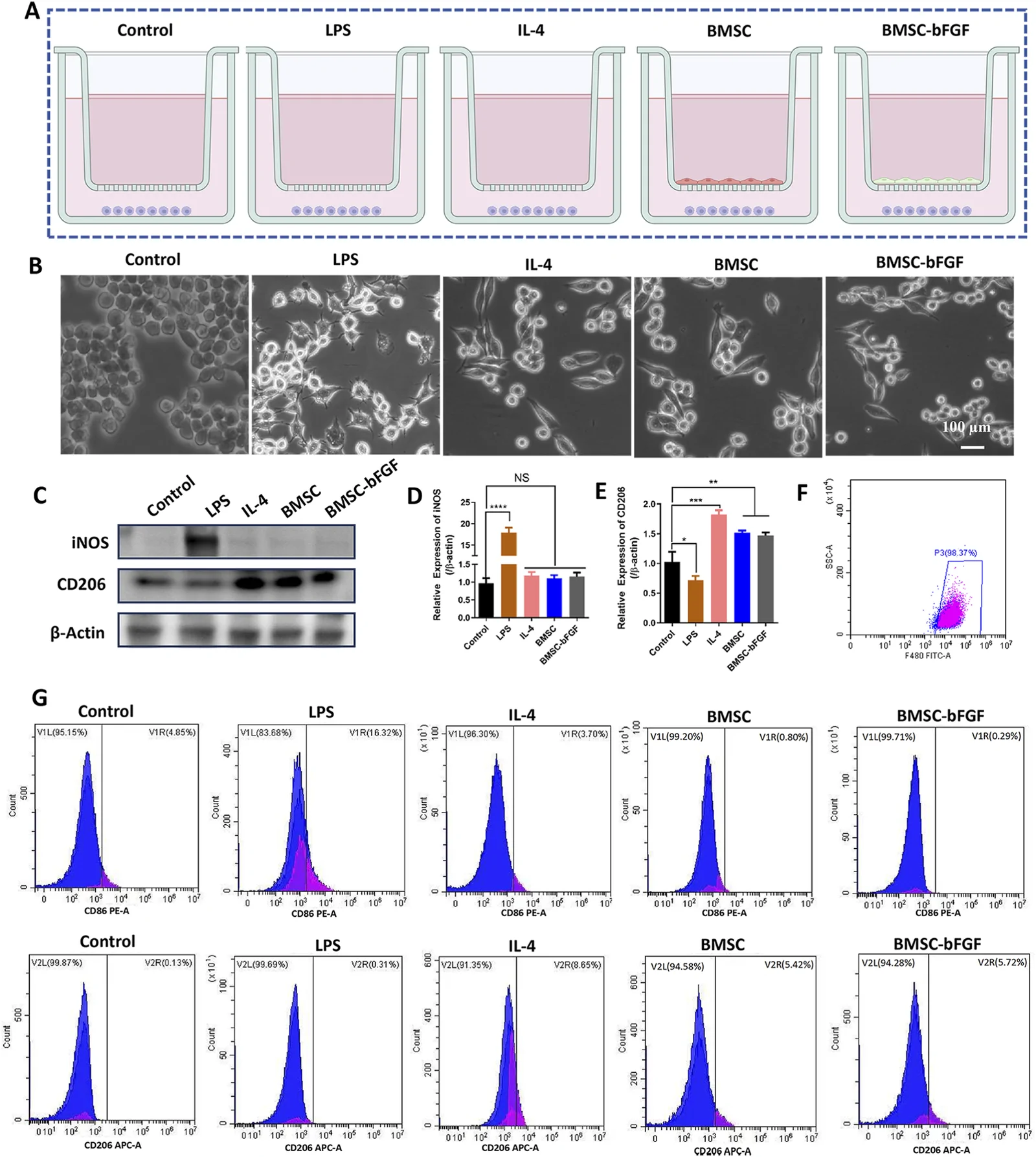
Immunomodulatory property of BMSCs. The indirect co-culture system. **(A)** Morphology of the macrophages in different stimulation conditions. **(B)** Scale bars = 100 μm. WB as well as semi-quantitative analysis of iNOS and CD206 expression after different treatments. **(C–E)** Evaluation of CD86 and CD206 expression of RAW264.7 cells after diverse treatment using flow cytometry. **(F,G)** Data are presented as mean ± SD. Significant differences are indicated as *p < 0.05, **p < 0.01, ***p < 0.005, ****p < 0.0001. NS, not significant.

### 3.6 Evaluation of fibrocartilage regeneration in rat tendon-to-bone injury

To investigate the effect of bFGF-transfected BMSCs on promoting fibrocartilage formation, a rat model of tendon-to-bone injury was established, and the regenerative efficacy was evaluated at 4 and 12 weeks post-surgery. Correspondingly, Hematoxylin and Eosin (H&E) and picrosirius red stainings were performed to observe the regenerated tendon-to-bone interface (TBI) healing histologically. As shown in Figures 4A,E,H staining revealed the presence of fibrovascular granulation tissue at the TBI in the BMSCs and BMSC-bFGF groups. In detail, macroscopic examination of the specimens showed no evident fibrocartilage regeneration in the control group at 4 weeks, whereas partial coverage by regenerated fibrocartilage was observed in the BMSCs and BMSC-bFGF groups (Figure 4A). At 12 weeks, newly formed fibrocartilage was observed in all groups, albeit considerable intergroup variability in both the regenerated area and quantity was detected (Figure 4A). As shown in Figure 4B, collagen arrangement in the BMSC-bFGF group was more uniform and organized than that in the other groups. More importantly, the ratio of reddish-yellow fiber area to total tendon area was higher in the BMSC-bFGF group, indicating the formation and maturation of well-oriented collagen fibers. Further, Safranin O-fast green (SOFG) and Toluidine blue (TB) stainings yielded results consistent with those of H&E staining (Figures 5A,B), where the area of metachromasia at the TBI healing site was larger in the BMSC-bFGF group than in the other two groups at both 4 and 12 weeks, and the lowest area was detected in the control group (Figures 5C,D).

**FIGURE 4:**
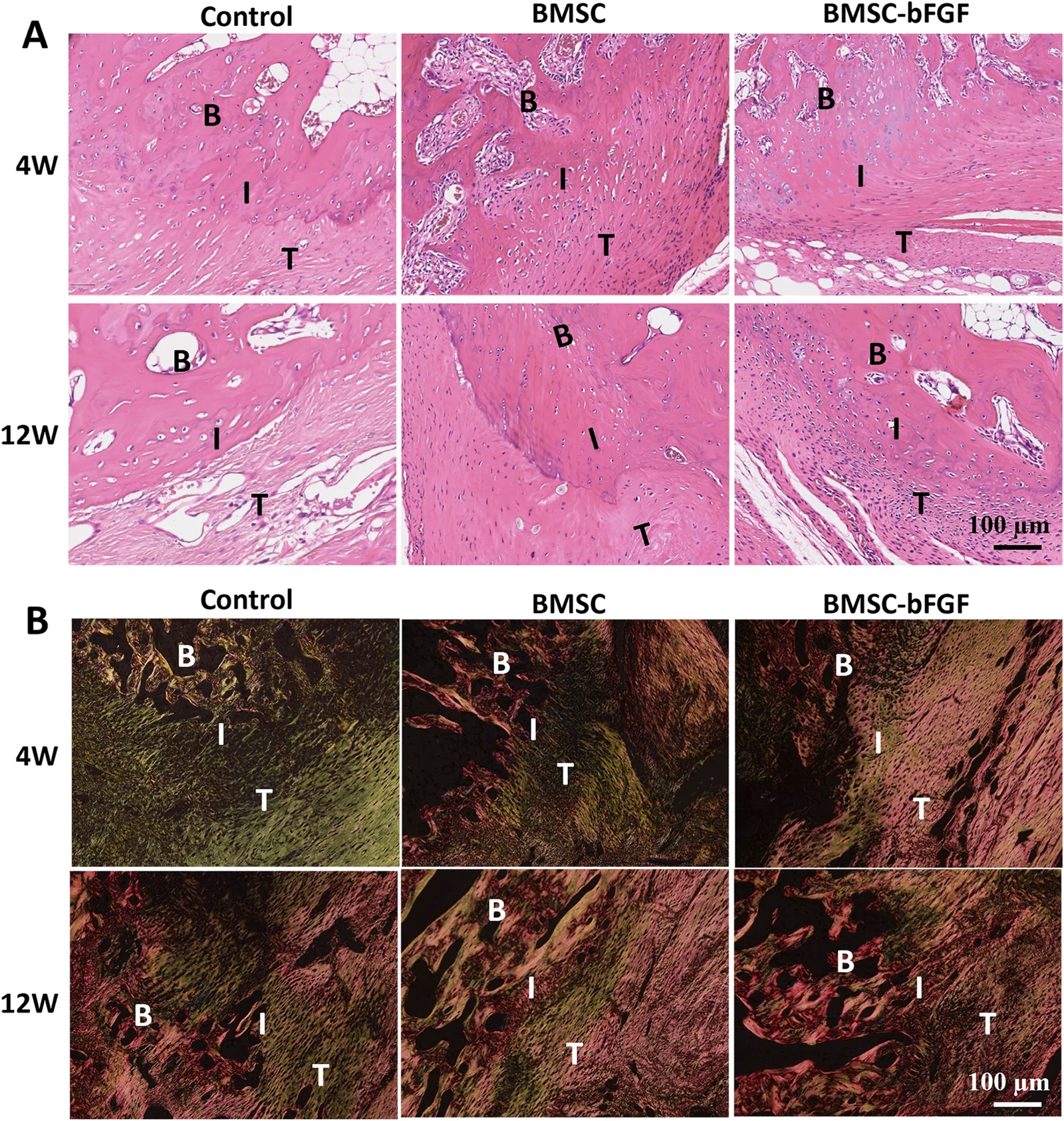
Histological assessment of the therapeutic efficacy of BMSCs-bFGF on TBI injury *in vivo*: **(A)** H&E staining of the repair region across TBI injury. **(B)** Picrosirius red–stained sections shown are under polarized light. Scale bars = 100 μm.

**FIGURE 5:**
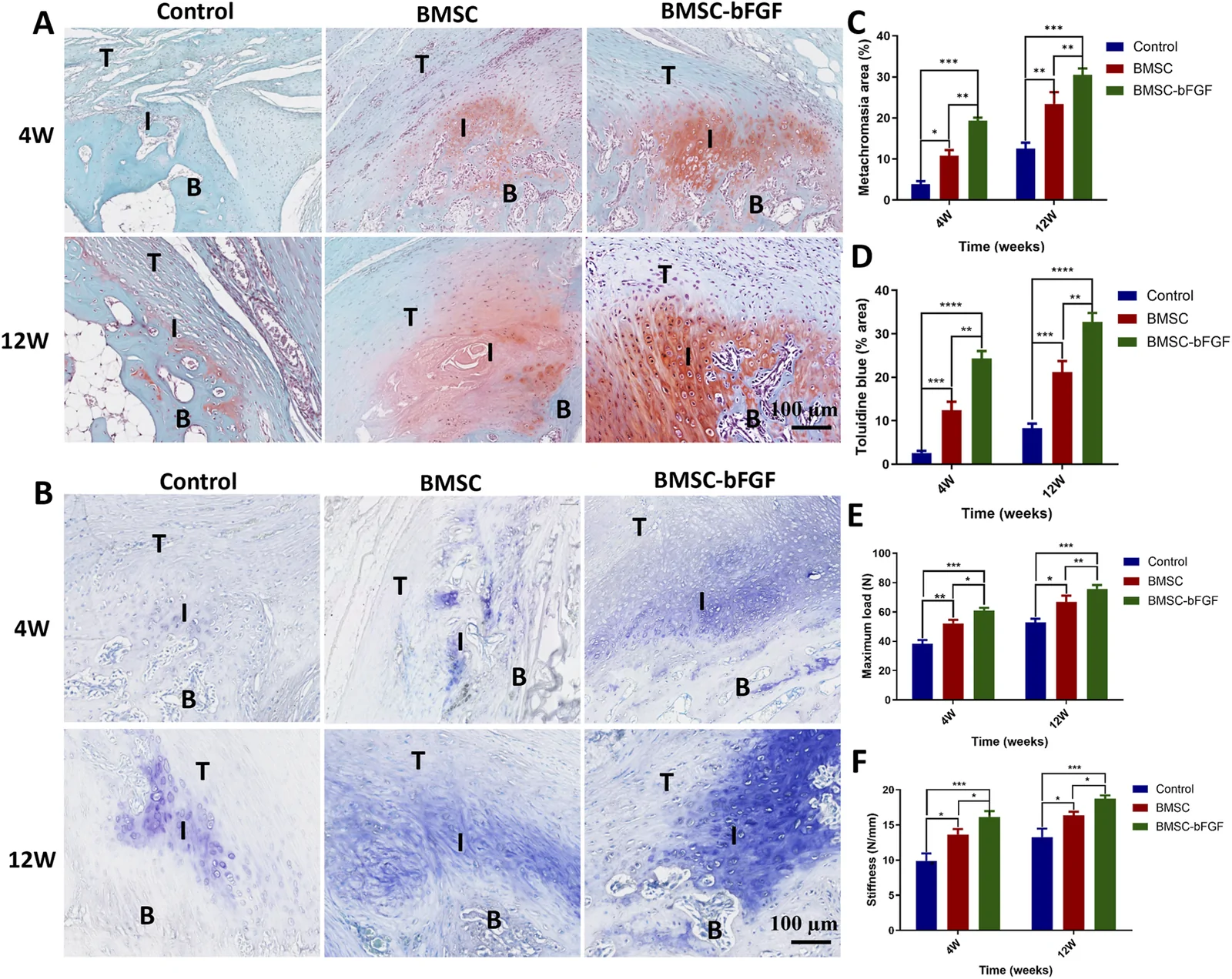
BMSCs-bFGF accelerated the recovery of TBI injuries *in vivo*: **(A,B)** Safranin O-Fast Green (SOFG) and toluidine blue (TB) staining of the repair region across TBI injury treated with different treatments. Quantitative analysis of the repair site across TBI injury treated with different treatments. **(C,D)** Results of maximum load and stiffness of the TBI after healing. **(E,F)** Scale bars = 100 μm. B, bone; I, interface; T, tendon. *p < 0.05, **p < 0.01, ***p < 0.005, ****p < 0.001.

### 3.7 Biomechanical performance evaluation

Biomechanical properties represent the ultimate metric for evaluating the regenerative quality of TBI healing. In this study, the maximum failure load and stiffness were selected as the biomechanical testing parameters. As shown in Figures 5E,F, both the maximum failure load and stiffness gradually increased in all groups from 4 to 12 weeks postoperatively. The maximum failure load in the BMSC-bFGF group was comparatively higher than that in each of the other two groups (Figure 5E). Similarly, the stiffness in the BMSC-bFGF group was also significantly greater than those of the other two groups (Figure 5F). At both time points, the maximum failure load and stiffness in the BMSC and BMSC-bFGF groups were significantly higher than those in the control group (all *p* < 0.05). Collectively, these findings indicated that the therapy of BMSC-bFGF greatly contributed to the favorable biomechanical performance of bone-to-tendon regeneration.

## 4 Discussion

The rapid recovery and full restoration of the enthesis function following TBI injury remain a significant clinical challenge. Even though bFGF, a kind of cytokine with chondrogenic property, was demonstrated to promote the chondrogenic differentiation of MSCs, whereas effective use of bFGF was hard to achieve due to the drawbacks of short half-life and rapid diffusion property (Zhao et al., 2007). In addition, high doses of bFGF could directly result in serious adverse reactions. Previous studies validated that viral vectors were the most efficient way for long-term gene delivery (Guan et al., 2015). To address these issues, mesenchymal stem cells (MSCs) were leveraged as a vector to carry bFGF genes by lentivirus transfection in this study. Nowadays, stem cell-based cell therapies have received more and more attention for their multifaceted role in tissue regeneration. BMSCs are a representative type of multipotent stromal cell that can differentiate into tenogenic lineages, osteogenic, and chondrogenic, under certain induction conditions. More importantly, BMSCs have been found to possess excellent immunomodulatory properties, enabling them to regulate immune cells, such as macrophages, to secrete anti-inflammatory cytokines (Zou et al., 2024; Peng et al., 2025; Long et al., 2026). These properties allow BMSCs to reduce excessive inflammation and facilitate the formation of a more functional enthesis-like structure. Furthermore, BMSCs lack specific surface markers, do not express MHC-II molecules, and exhibit low expression of major histocompatibility complex (MHC)-I molecules (Zou et al., 2024; Long et al., 2026). In addition, they do not express the molecules required for T lymphocyte activation (Zou et al., 2024) [11]. This characteristic endows BMSCs with potent immunoregulatory properties and low immunogenicity. Furthermore, upon transplantation of those cells into the body, they do not elicit a significant rejection response and can be allografted without triggering immune rejection (Han et al., 2019).

Recently, studies have shown that the disturbance of immune microenvironment played essential roles in tissue engineering and regenerative medicine (Butenko et al., 2024; Li Y. et al., 2025; Zhai et al., 2025). The participation of immune cells, especially the macrophages, are validated to be indispensable in determining the outcome of inflammation and tissue repair (Nie et al., 2024; Li Y. et al., 2025). As reported, macrophages can be typically classified into M1 and M2 phenotypes based on their pro-inflammatory and anti-inflammatory properties (Nie et al., 2024). Notably, a number of previous researches have shown that the M2 macrophages play a favorable role in the regeneration of injured tissues, while uncontrolled pro-inflammation by prolonged and excessive activation of macrophages can lead to extracellular matrix disorganization, scar formation, and biomechanical weakness at the injury site (Zhang et al., 2024) [8]. As reported, precise intercellular crosstalks between immune cells and MSCs are discovered to be central for tissue repair (Qi et al., 2018). MSCs secrete various cytokines, such as TGF-β and IL-10, which drive macrophage polarization towards the anti-inflammatory M2 phenotype, and suppression of inflammatory cytokines and signaling pathways, such as TNF-α, IL-1β, and IL-6, was also observed (Zou et al., 2024). In the present study, we found that the coculture of BMSCs and macrophages promoted the polarization of macrophages towards the M2 phenotype, which causally contributed to the regression of inflammation and clearance of debris.

The physiological healing of the injured TBI follows a sequential process analogous to that observed in other tissues, encompassing three distinct phases: inflammation, proliferation, and remodeling (He et al., 2024). Over recent decades, the critical role of the inflammatory phase has been extensively documented across diverse tissue repair and regenerative contexts, where the main role of early-stage infiltrated M1 macrophages is discovered to phagocytose pathogens and clear cellular debris for a long time period. However, the activation of M1 macrophages also possess conducive role to recruit stem cells by secreting chemokines, thereby facilitating TBI healing. To be noted, excessive secretion of inflammatory mediators aggravates local inflammatory microenvironment, debilitating the healing quality of TBI injury. What’s worse, the persistent activation of M1 macrophages at the healing site stimulates the fibroblasts to secrete excessive extracellular matrix (ECM), a main culprit to be blamed behind the formation of scar tissue (Gelberman et al., 2017). Therefore, sequentially transition of M1 to M2 macrophages were necessary and valuable for TBI injury, as M2 macrophages produce anti-inflammatory factors (such as IL-10 and TGF-β), which attenuates local inflammatory responses and accelerates tissue regeneration (Murray et al., 2014). In addition, macrophages secreted TGF-β3 is also a key regulator for chondrogenic differentiation of MSCs *in situ*, which profoundly fortifies fibrocartilage formation during TBI healing (Qu et al., 2019).

Our findings in this study demonstrated that BMSCs promoted TBI healing in a rat model by inducing the polarization of M0 toward M2 macrophages, where increased accumulation of anti-inflammatory cytokines and secreted regenerative growth factors were observed at the tendon-to-bone interface, which causally contributed to the earlier formation of fibrocartilage in such injury (Gao et al., 2022; Li et al., 2024). Moreover, to potentiate the chondrogenic differentiation of BMSCs, bFGF was transfected into BMSCs by lentivirus. To achieve the best transfection efficiency, bFGF gene transfection was attempted within an MOI range of 0, 50, 75 and 100, where an MOI of 75 obtained 93% gene transfection efficiency, the highest expression among all the predetermined parameters. After transfection, BMSCs observed the highest protein expressions of bFGF by Western blotting in such condition. This strategy not only preserved the capacity to modulate inflammatory response after TBI, but also promoted TBI healing via improving chondrogenic differentiation. Our results showed that the combination of BMSCs and bFGF significantly enhanced the healing quality of TBI healing, which provided novel insights and strategic directions for applying gene therapy and immunotherapy for treating tendon-to-bone injuries. As demonstrated, lentivirus transfection facilitated sustained and stable gene expression of bFGF in BMSCs, where BMSCs successfully observed chondrogenic differentiation owing to the enhanced expression of the bFGF protein in those cells. Despite the employment of a self-inactivating viral vector system, the potential risk of insertional mutagenesis cannot be entirely precluded. Consequently, further refinement and optimization of the vector design are imperative prior to its clinical translation.

To be noted, certain limitations were existed in this study. Firstly, although we found that macrophage was involved in the treatment of BMSC-bFGF in TBI healing, this was still an observational study, which lacks of investigation of the mode of action. Secondly, in addition to macrophages, numerous other immune cells, such as T lymphocytes and B lymphocytes may also play significant roles in TBI healing, however, such cells have not been studied. Thirdly, macrophage can be subdivided into several different phenotypes according to their function. But to simplify our research, we merely classified macrophages into M1 and M2 phenotypes. Therefore, the growth factors secreted by macrophages and the interactions between various inflammatory factors, as well as the temporal and spatial of the appearance of different macrophage phenotypes during TBI healing, should be in-depth studied in the future. Despite these limitations, our study indicates that BMSC-bFGF may provide an effective strategy for promoting TBI healing.

## 5 Conclusion

Our study demonstrated that the chondrogenic potential of BMSCs could be significantly enhanced by bFGF gene transfection. *In vivo* fibrocartilage formation assays showed that lentiviral-bFGF-transfected BMSCs provided sufficient stimuli for fibrocartilage formation, as these cells not only robustly potentiated their own chondrogenic capacity but also reversed inflammation by polarizing *in situ* macrophages from the pro-inflammatory M1 phenotype to the anti-inflammatory M2 phenotype, thereby critically contributing to the rapid recovery of TBI injury and excellent biomechanical performance. This study provides theoretical and experimental evidence supporting gene therapy for TBI injury.

## Data Availability

The raw data supporting the conclusions of this article will be made available by the authors, without undue reservation.
